# Correspondence between Dr. Knowles & Dr. Clarke

**Published:** 1807-04

**Authors:** Charles Murray

**Affiliations:** Bedford Row


### *To the Editors of the Medical and Physical Journal.*

GENTLEMEN,

THE Board of Directors of the Royal Jennerian Society, will be obliged to you to insert the following correspondence, which took place between Dr. Knowles, Secretary to the Medical Council, and Dr. Clarke of Nottingham, in consequence of the latter having expressed his disapprobation of the insertion of a letter, addressed by him to the Royal Jennerian Society, in Number lxxxvii. p. 426, of your Journal.

I am, &c.

*Bedford Row, March* 18, 1807. CHARLES MURRAY.

---

" To Dr. CLARKE, Nottingham.

" SIR,

" THE Medical Council of the Royal Jennerian Society for the Extermination of the Small-pox, deem it incumbent on them to apologize to you for their apparent inattention to your complaint against their late Secretary, in the Medical and Physical Journal for May ; owing to various unavoidable causes, which it would be useless to enumerate.

" They cannot, however, but express the surprise and concern which they felt, on seeing the comments of their late Secretary on your Communication, addressed to him in his official capacity ; a communication, which ought not to have been published without your knowledge and consent.

" They consider this act as highly unwarrantable, as a breach breach of trust, and a violation of duty; and the more to be regretted, as it tends to discourage that correspondence, which is so indispensably necessary for the accomplishment of the great object in which they are engaged.

" They do not coincide in opinion with their late Secretary, that a puncture in inoculation can be made too slightly, provided it produce infection; or, that inflammation, induration, or tumefaction of the arm, affords complete protection. When the progress of vaccination is interrupted, they recommend that the patient should be re-inoculated. Had their Secretary discovered any new criterion, he should have submitted it to their consideration, before he published it to the world; since their professional and public characters must, in some measure, be identified with his; and they could not but partake in any discredit that might be attached to him.

" But such a previous communication of his opinions was the more necessary to be made to the Medical Council, when his opinions were in direct opposition to those of the Council, deliberately drawn up, sanctioned by the Society at large, and published in their Instructions.— Those instructions, together with the advanced state of improvement to which Vaccination has now arrived, and particularly your own valuable Statement of the result of Inoculation, in those cases where the pustule was ruptured at an early period, will, it is hoped, prevent any detriment from ensuing, in consequence of the promulgation of opinions which they deem erroneous.

" The Medical Council therefore conclude, with expressing their warm approbation of the Plan of your Institution; the sincere pleasure which they feel at your success, and their anxious hope that nothing which has occurred will prevent them from being honoured with your future correspondence.

" By Order of the Medical Council.

*January* 26, 1807. " J. S. KNOWLES, Med. Sec."

---

" To J. S. KNOWLES, M. D. Secretary to the Royal Jennerian Society, London.

" SIR,

" I WAS certainly much dissatisfied with the conduct of the late Secretary to the Royal Jennerian Society, in publishing a letter, addressed to him, in his official capacity, evidently not prepared for that purpose, but written in a confidential manner, to gain information, in the first first establishment of our Institution, to remove our impediments, and to facilitate our practice.

" I was, at that time, more particularly anxious to receive the advice of the Medical Council; differing as I did in opinion upon some few points with my Medical Brethren, I became in some measure responsible for any deviations from the usual practice, and of course more desirous to have my opinions either confirmed or corrected from an authority, so justly valued.

" I shall not here make any comment on the self importance, assumed on the above occasion, in making annotations on my communication; or the illiberality with which the late Secretary treated all who might presume to differ from him in opinion.

" The very handsome and liberal manner, with which the Society has communicated to me its sentiments on the occasion, demands my warmest thanks; nor did I for one moment suspect, that it ever could give sanction to conduct, so immediately destructive of confidence, so inimical to medical inquiry.

" I must likewise express my satisfaction at the candour which pervades the whole communication, and my firm conviction, that experience has already proved the absolute necessity of a second inoculation, when the progress of vaccination is interrupted.

" I am requested to thank the Society for the flattering approbation of the Plan of our Institution.

" The characteristic, assumed by your late Secretary, has, in some places, been thought very satisfactory; such confidence may, in future time, be productive of great injury to vaccination; but it dimiuishes the trouble of the practitioner, increases his practice, and, for a time, gives him the decided advantage over the more cautious and timid inoculator.

" Yet, the time will come, when the veil will be thrown aside, and that dreadful monster, the small-pox, begin his ravages; when many of those unhappy persons, now smiling in thoughtless security, may fall the victims to a fanciful hypothesis.

" We have not been informed of an instance of small-pox since January 1806, nor do I believe one case has been seen. This may be thought very satisfactory, but it has produced so much apathy, or fancied security, among the poor, that they think vaccination totally unnecessary; not finding the enemy at their door, they do not conceive it necessary to guard against him. The probable consequence of this folly is too evident; should the small-pox make make its appearance, we shall certainly make every exertion to stop its progress; at present, it is with much difficulty we can answer the demands for vaccine virus.

"I must again express my sincere thanks for your very polite letter, and beg you would personally accept my grateful acknowledgements, for the handsome manner in which you have conveyed the sentiments of your Society.

"I am, &c.

*Nottingham, Jan.* 8, 1807. "JAMES CLARKE."

P. S. Should the Society express any wish to publish your letter with my answer, I can have no objection; it may have the good effect of destroying any unfavourable impressions, if any such have been excited, by the conduct of the late Secretary.

